# Chinese nurses' perceptions on toxic leadership behaviours of nurse managers: A qualitative study

**DOI:** 10.1111/jonm.13758

**Published:** 2022-08-23

**Authors:** Xueqin Guo, Lijuan Xiong, Yumei Wang, Xin Li, Yuhan Wang, Fang Xiao, Jia He, Yuting Xiang, Chenzi Xu

**Affiliations:** ^1^ Union Hospital, Tongji Medical College Huazhong University of Science and Technology Wuhan Hubei Province China; ^2^ School of Nursing, Tongji Medical College Huazhong University of Science and Technology Wuhan Hubei Province China

**Keywords:** abusive supervision, interview, nurse manager, qualitative, toxic leadership

## Abstract

**Aim:**

The aim of this study is to explore the perceptions of Chinese registered nurses on toxic leadership behaviours of nurse managers and to determine its type, cause and response measures.

**Background:**

The nurse manager is the front‐line leader of the nurses who provide services directly to patients. Previous evidence suggests that toxic leadership behaviours of nurse managers do exist and it is necessary to understand the specifics of it.

**Methods:**

We used phenomenological research methods to conduct semi‐structured in‐depth interviews among 12 nurses at a tertiary hospital in Wuhan over the period from January to March 2022. And the data were analysed using Colaizzi seven‐step analysis method.

**Results:**

Four themes were discovered: (a) nurses' perceptions of toxic leadership behaviours; (b) toxic leadership behaviours of nurse managers; (c) reasons for toxic leadership behaviours of nurse managers and (d) measures for toxic leadership behaviours of nurse managers.

**Conclusion:**

Chinese nurses are exposed to the toxic leadership of nurse managers for multiple reasons and respond differently.

**Implications for Nursing Management:**

This study helps nursing managers identify which behaviours are harmful to the nurse that require special attention in developing strategies to buffer against nurse managers' toxic leadership.

## 1 INTRODUCTION

Leadership behaviour has always been the focus of management research. Most of the past investigations have focused on positive leadership behaviours such as transformational leadership and sincere leadership but paid less attention to negative leadership behaviours. Toxic leadership behaviour, as a negative and ineffective leadership behaviour, does exist in nursing and is becoming more common (Labrague, 2021). In the workplace, the leadership of the nurse managers has an impact on nurses, patients and organizations. Research repeatedly reports the consequences of toxic behaviours in terms of poor psychological health (Zhang et al., 2021), low job satisfaction, low quality of nursing care (Sharif et al., 2021), counterproductive work behaviours (Low et al., 2021) and higher turnover intention (Lyu et al., 2019). As to patients, the incidence of nurse‐reported adverse events increased, such as medical errors (Lyu et al., 2019) and the decline in care quality (Labrague, 2021). To the organization, the reduced organizational performance and the emergence of toxic followers can lead to the breeding of negative organizational culture (Thomas et al., 2016). The existence of the toxic leadership behaviours of nurse managers is widely concerned.

Prior studies, mainly in western countries, have identified its definition, prevalence, classification, causes and effects in different cultural backgrounds. However, there is little known about the toxic leadership behaviours of nurse managers in Chinese hospitals. Chinese hospitals mainly advocate positive leadership behaviour such as transformational leadership styles but pay less attention to negative leadership behaviour. Therefore, this qualitative research explores Chinese nurses' perceptions of toxic leadership behaviours of nurse managers and identify the type, cause and response measures.

## 2 BACKGROUND

Past research has attempted to identify nonviolent harmful behaviours among health care leaders. Concepts involving such behaviours include abusive management, petty tyranny, workplace bullying and toxic leadership (Guo et al., 2021). There is theoretical and empirical overlap between these concepts. Toxic leadership refers to the use of organized, systematic and persistent destructive behaviours by leaders that may cause psychological harm to their followers and harm to the organization, emphasizing undesirable outcomes (Webster et al., 2016). Unlike workplace bullying (Tuna & Kahraman, 2019), which is repeated hostile behaviour of one person or group towards another, toxic leadership behaviour specifically refers to behaviour from managers to subordinates and does not include bullying from colleagues.

In recent years, many studies have pointed to the presence of toxic leadership, which has become prevalent in nursing and other health care professions (Labrague, Nwafor, & Tsaras, 2020; Zhang et al., 2021). Filipino nurses rated their nurse managers' toxic leadership behaviours to a lesser extent, suggesting that Filipino nurse managers have a lower tendency to exhibit toxic leadership behaviours (Labrague et al., 2021). A survey of 224 nurses in Turkey showed that nurses were exposed to a moderate number of toxic leaders. The overall mean score of the Toxic Leadership Scale was 78.64 ± 29.24 with a total score between 30 and 150 (Ozkan et al., 2022). These studies indicate that nurse leaders may exhibit toxic behaviours in the treatment of subordinates in the nursing field.

Toxic leadership is characterized by chronic abuse of subordinates, including public ridicule, threats, deliberate withholding of needed information and silent treatment (Peltokorpi & Ramaswami, 2021). They always find ways to crush and reduce the confidence and interest of subordinates (Singh et al., 2018). They are destructive, abusive and ineffective, interfering with the ability of others to do their jobs. They are concerned with status and power, thus increasing the toxicity of the environment (Milosevic et al., 2020).

Toxic leadership can manifest in different ways in another context and therefore can lead to different outcomes (Milosevic et al., 2020). The Chinese work culture emphasizes harmony in collective relationships on the one hand. Managers need to be fair and selfless and care for subordinates. On the other hand, it requires managers to be strict in rewards and punishments and goal oriented. There is a large power distance and a clear organizational hierarchy in Chinese work (Sun et al., 2020; Yin et al., 2021). Chinese managers' leadership behaviours are strongly influenced by traditional Chinese culture (Barney & Zhang, 2009; Ma & Tsui, 2015). Therefore, toxic leadership in the Chinese cultural context may have different characteristics from other countries.

In China, nursing staff is roughly divided into three categories. The first is registered nurses who provide direct services to patients within the unit. The second is nurse managers, who directly supervise and direct the clinical work of registered nurses. The third is nursing department managers, who manage the nurse managers throughout the hospital. As a significant component of nursing management in hospitals in China, do toxic leadership behaviours occur in the management process of nurse managers? What are the specific types of toxic leadership behaviours perceived by nurses? Why does toxic leadership occur in the nursing workplace? How do nurses deal with these toxic leadership behaviours? The identification of these elements is critical to the healthy development of nursing management.

There is no theoretical framework to explain the manifestation of toxic leadership behaviour; the Toxic Triangle model (Padilla et al., 2007) can help to understand the development of it. In China, researchers used a case interview method, combined with literature analysis and rooting theory, to construct a ‘new’ toxic triangle model based on the toxic triangle model (Chen & Sun, 2021). It consists of three elements: supervisory traits, subordination factors and organizational context. Supervisor traits include narcissistic tendencies, power consciousness, outcome orientation, stereotypes and biases. Subordinate factors include self‐perception, behavioural style, job performance and political skills. Organizational contextual factors include power distance, Chaxu Climate, promotion channels and grievance mechanisms. Among them, the Chaxu climate emphasizes interpersonal relationships. ‘Cha’ represents the inequitable distribution of resources in the organization. ‘Xu’ represents the status gap between superiors and subordinates, emphasizing the order of respect and inferiority in the organization (Ma & Su, 2020). Supervisors will use the intimacy of interpersonal relationships as the main reference standard in resource allocation and job evaluation, losing quantitative fairness. This can result in affiliates and cliques in the organization. The combination of these three elements will promote toxic leadership.

## 3 METHODS

### 3.1 Design

We applied a descriptive, qualitative study using individual telephone semi‐structured interviews. The aim is to understand the toxic leadership behaviours of nurse managers perceived by nurses by revealing the reality and experience of repeated hostile behaviours at work. Find out its type, cause and response measures through further analysis.

### 3.2 Participants

Purposeful sampling was used to recruit nurses in a tertiary hospital in Wuhan, which has a total of 3,733 nurses on staff and 197 nurse managers. Inclusion criteria for interviewees possess a nurse's practice qualification, informed consent and voluntary participation. Exclusion criteria: nurses who experienced a bad psychological impact event (e.g. divorce, death of an influential relative or sick leave) within three months, and nurses in internship. The rationale for including the target population for this survey was that these nurses were managed by nursing managers and provided direct patient care. Nurses with their psychological distress were excluded to minimize the influence of other factors. The demographic profile of participants is shown in Table 1.

**TABLE 1:** Demographics of nurse participants (*N* = 12)

Characteristics	*n*	%
Education		
Bachelor's degree	8	66.7%
Master's degree	4	33.3%
Marital status		
Married	5	41.7%
Single	7	58.3%
Age, mean = 32.01, SD = 6.50		
20–30	6	50.0%
31–40	4	33.3%
41–50	2	16.7%
Years as nurse, mean = 9.92, SD = 8.30		
0–10	7	58.3%
11–20	3	25.0%
21–30	2	16.7%
Nurse career title level		
Junior nurse	2	16.7%
Junior nurse practitioner	4	33.3%
Supervising nurse practitioner	5	41.7%
Associate chief nurse practitioner	1	8.3%

*Note*: *n* = number of nurses.

### 3.3 Data collection

This study was conducted from January to March 2022 using semi‐structured, in‐depth, open‐ended and recorded interviews. To create a relaxed atmosphere, it began with light topics such as personal information and expectations for ideal leaders. Before the formal interview, respondents were asked to recall specific instances of toxic leadership behaviours of nurse managers that they or their colleagues had experienced. The researcher asked appropriate follow‐up questions, listened carefully and took notes. The detailed interview outline is shown in Table 2.

**TABLE 2:** Interview questions

Please briefly introduce yourself (age, marital status, time spent independently in nursing, nursing title)
What is the personality and work style of the head nurse on your unit? How do you feel this work style has contributed to your professional performance?
Have you heard of toxic leadership behaviours of nurse leaders? What behaviours do you think are toxic for nurse leaders?
Have you yourself or a colleague ever suffered from toxic nurse leader behaviour? What was the situation at the time?
Why do you think nurse leaders exhibit this toxic behaviour?
What are the effects of this nurse leader toxic behaviour?
What would you do if your nurse leader exhibited toxic behaviour?
Do you know of any countermeasures or ways to respond to or address negative leadership behaviours of nurse leaders in your hospital?

*Source*: Primary author.

Due to the sensitivity of the subordinate relationship between nurses and nurse managers, respondents may not be able to express their true thoughts fully during face‐to‐face conversations. Consequently, the interviews were conducted by telephone. The anonymity of telephone contact enables respondents to be more proactive in answering questions without feeling constrained (Musselwhite et al., 2007) so that it can be considered an appropriate method of data collection (Ward et al., 2015). Data collection ended when the conceptual information achieved saturation and no new data emerged (Saunders et al., 2018).

### 3.4 Data analysis

The mean duration of interviews was 38 min, ranging from 28 to 59 min, and each was transcribed within 24 h of the interview. We used NVIVO11.0 computer software to facilitate the analysis of interview transcripts. The data were analysed by Colaizzi's (1978, p. 48–71) seven‐step method. First, two authors reviewed transcribed interviews several times and had a general understanding of the whole content. Second, they analysed the data word by word to identify and extract significant statements related. In the third step, they determined and formulated meanings from substantial statements. Then, they look for common concepts and organize formulated meanings into categories and clusters of themes. In the fifth step, they exhaustively describe the investigated phenomenon by adding typical original statements from participants. Next, they put similar themes and their descriptions together for repeated comparisons to draw similar views and construct themes. Finally, returning the resulting subject structure to the participants to check, if there is a bias, the researcher must step back to the analysis from the first step.

### 3.5 Rigour

The following measures have been taken to ensure the consistency and validity of the study. The researchers have clinical experience that can easily communicate with the nurses and build trust. The researchers ensured that the interviewees correctly understood toxic leadership behaviours before the interview. The nominal themes and subthemes identified by the two researchers were discussed and agreed. All respondents were invited to confirm the distilled results, and they agreed with the findings.

### 3.6 Ethical considerations

This study protocol was approved by the Ethical Committee of Tongji Medical College, Huazhong University of Science and Technology (No. S044), and followed the ethical principles of the Declaration of Helsinki. Before the beginning of each interview, participants were informed verbally, and in writing about basic information including the purpose, significance, the principles such as voluntary participation, confidentiality and anonymity, the need for audio recording and the experience of recalling might be psychologically uncomfortable. They could terminate and discontinue at any time in the study.

## 4 RESULTS

We derived 4 main categories and 15 sub‐categories from the data. They are described in Table 3. The following interpretations and direct quotations (translated from Chinese) describe the meaning of each theme in order to be representative of the nurses' views.

**TABLE 3:** Main categories and subcategories of the study

Main categories	Sub‐categories
Nurses' perceptions of toxic leadership behaviours	Hard to avoid
	Desire for attention
Toxic leadership behaviours of nurse managers	Negative feedback
	Ignoring
	Unfair treatment
	Self‐centeredness
	Excessive pressure
	Work inaction
Reasons for toxic leadership behaviours of nurse managers	Workload
	Personality
	Job performance
Measures for toxic leadership behaviours of nurse managers	Confusion and silence
	Department or industry change
	Reflection and communication
	Flatter and ingratiation

### 4.1 Theme 1: Nurses' perceptions of toxic leadership behaviours

#### 4.1.1 Hard to avoid

Most respondents consider that toxic leadership is inevitable in the workplace. A nurse said: ‘I know toxic behavior is bad, but the workplace will have it, wherever you go’ (N4). Another nurse said: ‘This thing is very common, no matter what the manager does, there will be people who are not satisfied’ (N6).

#### 4.1.2 Desire for attention

Respondents said that in the face of toxic leadership behaviour, everyone will selectively see only the positive side. They hope to get enough attention for the negative side. A nurse said: ‘…people will not bring out the toxic leaders to discuss a specific solution’ (N3). Another nurse said: ‘The hospital asks nurses to evaluate the nurse manager, every time this happens, we will give a good score, even if it is hypocritical’ (N7). A nurse explained: ‘The nurse manager has some toxic leadership behaviors, but she must have a positive side, otherwise how could she be elected as a manager? Everyone tends to say good things about managers’ (N11).

### 4.2 Theme 2: Toxic leadership behaviours of nurse managers

#### 4.2.1 Negative feedback

All respondents desired care and understanding at work, as well as verbal evaluations and constructive feedback from their nurse managers. They claimed that certain nurse managers frequently utilized negative feedback to illustrate that the nurses' performance was inadequate or inappropriate and to dismiss and criticize the nurses' personal and professional accomplishments. A nurse recalled, ‘Once I carefully provided an infusion, the nurse manager told me I was wrong in front of the family members of patients and reprimanded me harshly. After that, she kept telling me what I had done wrong’ (N2). Furthermore, ‘Our supervisor does not point out your mistakes in your presence, but she blames you in front of other nurses or doctors in the department’ (N5). Another nurse talked about the interaction with her head nurse at work, ‘She would make some bad facial expressions to you, like rolling her eyes or glaring at you’ (N7).

#### 4.2.2 Ignoring

Half of the nurses had been neglected by their leaders. They described toxic leadership practices as nurse leaders' scorn and indifference to nurses in the unit. Some nurses went into detail about situations. ‘When I have a conflict with a patient, the nurse manager does not listen to my explanation and just makes me apologize to the patient’ (N1). One nurse explained, ‘Our chief nurse rarely appreciates us and continually stresses that if you are not content, you may quit. More people can replace you no matter how good you are’ (N3).

#### 4.2.3 Unfairness

Some interviewees claimed that even though they worked in the same department, the administrator treated them unfairly for various reasons. N4 expressed this unfair treatment: ‘You can be blamed and deducted for the same mistake while others get nothing’. Another nurse said, ‘Accountability team assignments are unfair. You always have the most work to complete, and your two days off on the night shift are sometimes purposely separated’ (N10).

#### 4.2.4 Self‐centeredness

According to several respondents, nurse leaders can demonstrate self‐centred traits such as narcissism and selfishness. ‘The head nurse will actively highlight her leadership position in front of the nurses, exhibit a sense of superiority and give us a strong sense of remoteness’, one nurse stated (N6). ‘You have to consult the chief nurse to make any decision in the department. Moreover, do not argue with her. Otherwise, you will be required to self‐examine’, one nurse explained (N9). One nurse mentioned, ‘The chief nurse admires to be complemented by the nurses. She thinks that everyone in the unit is around her for cajoling her and making her available’ (N11). In addition, one nurse shared, ‘Our head nurse often asked us to do things but would announce to the public that she did these things herself …’ (N13).

#### 4.2.5 Excessive pressure

Respondents noted that nurse managers frequently assign many non‐repetitive tasks at work and add exert pressure. One nurse exclaimed, ‘The nurse manager is part of the unit and requires nurses to do research and publish articles, but we do not have the foundation or resources to learn …’ (N3). One nurse said she faced a similar dilemma: ‘Nurses are asked to clean up every day, and we do not know if we are cleaners or what’ (N6). Another nurse expressed her experience in the following words: ‘Departmental training, exams, and inspections are widespread, and even all exam papers are also required to be memorized or else you will have your salary deducted’ (N7).

#### 4.2.6 Work inaction

We also received information from the interviewees that the head nurse was inactive in her work. For example, ‘The head nurse ignores conflicts between nurses when they arise’ (N8). One nurse explained her situation in these words: ‘Once the unit was very busy and needed to be coordinated, so I reported it to the head nurse. But the head nurse told me to find my way and asked me to find my colleagues to work overtime’ (N11).

### 4.3 Theme 3: Reasons for exposure to toxic leadership behaviours of nurse managers

#### 4.3.1 Workload

The vast majority of interviewees described the reason for the toxic leadership behaviours of a nurse manager as a lack of labour that included not only nurses but also the nurse managers. One interviewee revealed that ‘patients and nurses are both under the charge of the nurse manager; although she does not have to care for patients, she has to do research, publish articles, go to meetings, etc’ (N2). Another nurse pointed out that ‘the department is understaffed, and there are only a few nurses working and even fewer on the night shift’ (N5).

#### 4.3.2 Personality

Personality was identified as a cause. The personality of the head nurse is described as ‘… low emotional intelligence, tending to reopen old scores … narrow‐minded, and not wanting subordinates to develop better’ (N1). ‘… competitive and doing everything to the best to emphasize her ability’ (N11). A few nurses mentioned that nurses' personality also affects whether they would be exposed to toxic leadership. ‘Nice guys finish last, and nurses with weak personalities will be bullied by their leaders’ (N7), and ‘Some nurses are too opinionated to obey the arrangement of their leaders’ (N3).

#### 4.3.3 Job performance

Some respondents believed that nurses' performance leads to toxic leadership, and the respondents shared their story that ‘if you are negligent and make mistakes frequently, of course, you will always be supervised’ (N6). Respondents reported their experience that ‘they do not do their daily nursing work well enough to meet the leader's demands’ (N4).

### 4.4 Theme 4: Measures for toxic leadership behaviours of nurse managers

#### 4.4.1 Confusion and silence

When asked about responding to the toxic leadership behaviours, the majority of nurses said they were overwhelmed with silence and would not respond. A nurse uttered, ‘I have no idea what to do … Even if you report it, it will not change anything, and it will just make the leader dislike you more’ (N1). Another nurse mentioned that ‘when encountering such an incident, our department would rather work on the night shift than the day shift in order to work without the head nurse. I will do my best to forget the bad experience and avoid interacting with the chief nurse in the workplace’ (N5).

#### 4.4.2 Department or industry change

Most of the nurses pointed out that they can choose to resign or work in different departments in the same hospital to escape from their current environment. For example, one nurse stated, ‘Do not do nursing without a strong heart and an iron body, or you will think about resigning every day. Moreover, many people in our department were resigned because the head nurse’ (N2). Another nurse answered, ‘I can change my department and living environment’ (N8).

#### 4.4.3 Reflection and communication

Some nurses reported that they would self‐reflect after receiving toxic leadership from the nurse manager, try to communicate with the nurse manager and then make changes from themselves. ‘First of all, I would reflect on myself and think why it was me. If I made a mistake, I would correct it next time’ (N7). Another nurse responded, ‘If you have a problem with the head nurse, you should communicate with her/him directly instead of hiding it in your heart, which will only increase your psychological burden’ (N11).

#### 4.4.4 Flatter and ingratiation

A few nurses mentioned that after receiving toxic leadership from the nurse manager, you just need to do ‘little things’ to change your situation, such as ‘saying what the head nurse likes to hear, learning to read people's minds, and kissing ass’ (N12) as well as ‘doing as she pleases’ (N4).

## 5 DISCUSSION

This article analysed nurses' perceptions of toxic leadership behaviours among nurse managers, and these open‐ended responses replenish the missing piece of the quantitative problem. We know that toxic leadership does exist in the workplace of Chinese nurses, and nurses want to draw attention. Toxic leadership is not limited to one country. It is a global problem.

Previous studies have shown that nurse leaders' toxic behaviours are multifaceted. Labrague, Lorica et al. (2020) categorized the toxic leadership behaviours of nurse leaders into four types of behaviours: intemperateness, narcissism, self‐promotion and humiliation. In addition to these apparent behaviours, toxic behaviours of leaders sometimes involve covert and passive behaviours, such as the failure to support staff and the constant use of insulting and offensive nonverbal gestures (Milosevic et al., 2020). The specific manifestations of toxic behaviours of Chinese nurse leaders in this study included excessive negative feedback, neglect, differential treatment, pressure on subordinates, self‐centeredness and inaction at work. In contrast to foreign countries, supervisors rarely displayed pronounced superficial ‘hostility’ when they disliked or blamed an employee, such as physical aggression or uncontrolled behaviour. Due to the high context of Chinese culture (Ding et al., 2016), leaders use covert and disguised ways to express such thoughts and feelings implicitly.

The new ‘toxic triangle’ framework (Chen & Sun, 2021) suggests that toxic leadership causes include supervisory traits, subordination factors and organizational context. Leadership behaviour is a leader–member exchange interaction, and the personal characteristics of both leaders and subordinates influence toxic leadership. Among supervisor traits and subordinate factors, except those mentioned in the toxic triangle, previous research has shown that leaders who are poorly managed (Gunawan et al., 2018), chronically psychologically depressed (Tepper et al., 2006) and sleep deprived (Barnes et al., 2014) are more likely to practice toxic leadership. Employees who dare to overcome their fears and adhere to their values and principles are less likely to be affected by toxic leadership behaviours (Afsar et al., 2019). This study concluded that the personalities of nurses and nurse managers may contribute to toxic leadership. Factors such as low emotional intelligence, narrow‐mindedness, goal‐orientedness of nurse managers, cowardice and stubbornness of nurses may also contribute to toxic leadership behaviours. Among the organizational context factors, since the power and authority of the nurse manager are not absolute or highly concentrated (Martinko et al., 2013), organizational factors mentioned in the ‘toxic triangle’ framework, such as power distance, Chaxu Climate, promotion channels and grievance mechanisms, as pointed out in other studies such as opaque promotion channels, which require a leader's recommendation to be promoted, are not evident in this study. In addition, workload factors are a major cause, the shortage of nurses leads to excessive work imposed by leaders on their subordinates and leaders produce toxic leadership to achieve desired goals, which is consistent with Labrague et al.'s study (2021).

Respondents indicated that the vast majority are in a state of helplessness after encountering toxic leadership. Some nurses choose to resign or change departments; in China, a survey of 28,910 nurses showed that 22.02% of nurses often have the idea of changing jobs recently. In the coming year, 70.80% choose to leave their dissatisfaction with their current jobs, and 76.53% choose not to continue their current careers after leaving (Wu et al., 2022). Some nurses who are reluctant to leave may become advocates to maintain and promote the rationalization of toxic organizational leadership (She, 2020). Organizations do not provide avenues for grievances and do not have good responses to toxic nurse leaders. Despite of extensive research and implementation of various organizational development and human resource interventions, it is unrealistic to expect leaders to change suddenly. Most importantly, leaders can be made aware of the harmful nature of the behaviour, and organizations can establish more systematic intervention and feedback systems.

## 6 LIMITATIONS

This study is based on nurses' perceptions of toxic leadership behaviours. Thus, the real frequency of toxic leadership behaviours of nurse managers at this workplace could not be determined. In China, nurses from different levels of hospitals are exposed to the toxic leadership behaviours of nurse managers. This study only recruited nurses working in tertiary hospitals. The scope of further study should be conducted to expand the sample's representativeness.

## 7 CONCLUSIONS

This study is an exploration of toxic leadership behaviours of nurse managers from the Chinese nurses' perspective. First, compared with other countries, the severity of toxic leadership behaviours of nurse managers among Chinese nurses was not reflected in the apparent hostile act with an uncompromising attitude but in some unfriendly, concealed and disguised behaviours. The causes are complex and deserve to be explore in‐depth. Finally, we found that nurses have different ways of responding, some of which can affect nursing care. The findings can help nurse managers determine which behaviours are harmful to nurses and respond at the source. The importance of oversight by more senior managers in the organization is emphasized to promote a positive work environment for nurses. We can no longer pretend that nothing is happening; we need to work to address this issue and protect the work environment for nurses.

## 8 IMPLICATIONS FOR NURSING MANAGEMENT

Nurse managers need to follow the principle of being strict with themselves and lenient with others and take the initiative to prevent the emergence of toxic leadership behaviours. Nurses urgently need to improve their ability to identify and respond to negative leadership behaviours, regulate their emotions and work with colleagues to create a harmonious departmental atmosphere. The organization should strengthen its care for nurse leaders, pay attention to and promptly discover negative leadership behaviours of nurse leaders, open up channels, clarify attitudes, find out the facts and deal with them seriously.

## CONFLICT OF INTEREST

All authors declare no conflict of interest.

## ETHICS STATEMENT

The study was approved by the Ethics Committee of Tongji Medical College, Huazhong University of Science and Technology (No. S044).

## AUTHOR CONTRIBUTIONS

The authors conceptualized and designed the study, conducted data collection and analysis and drafted and wrote the paper.

## Data Availability

Data available on request due to privacy/ethical restrictions.
